# Characterization of EvaGreen and the implication of its physicochemical properties for qPCR applications

**DOI:** 10.1186/1472-6750-7-76

**Published:** 2007-11-09

**Authors:** Fei Mao, Wai-Yee Leung, Xing Xin

**Affiliations:** 1 Biotium, Inc. Hayward, California, USA; 2 AlleLogic Biosciences Corporation, Hayward, California, USA

## Abstract

**Background:**

EvaGreen (EG) is a newly developed DNA-binding dye that has recently been used in quantitative real-time PCR (qPCR), post-PCR DNA melt curve analysis and several other applications. However, very little is known about the physicochemical properties of the dye and their relevance to the applications, particularly to qPCR and post PCR DNA melt curve analysis. In this paper, we characterized EG along with a widely used qPCR dye, SYBR Green I (SG), for their DNA-binding properties and stability, and compared their performance in qPCR under a variety of conditions.

**Results:**

This study systematically compared the DNA binding profiles of the two dyes under different conditions and had these findings: a) EG had a lower binding affinity for both double-stranded DNA (dsDNA) and single-stranded DNA (ssDNA) than SG; b) EG showed no apparent preference for either GC- or AT-rich sequence while SG had a slight preference for AT-rich sequence; c) both dyes showed substantially lower affinity toward ssDNA than toward dsDNA and even lower affinity toward shorter ssDNA fragments except that this trend was more pronounced for EG. Our results also demonstrated that EG was stable both under PCR condition and during routine storage and handling. In the comparative qPCR study, both EG and SG exhibited PCR interference when used at high dye concentration, as evident from delayed Ct and/or nonspecific product formation. The problem worsened when the chain extension time was shortened or when the amplicon size was relatively long (>500 bp). However, qPCR using EG tolerated a significantly higher dye concentration, thus permitting a more robust PCR signal as well as a sharper and stronger DNA melt peak. These differences in qPCR performance between the two dyes are believed to be attributable to their differences in DNA binding profiles.

**Conclusion:**

These findings suggest that an ideal qPCR dye should possess several DNA-binding characteristics, including a "just right" affinity for dsDNA and low or no affinity for ssDNA and short DNA fragments. The favorable DNA-binding profile of EG, coupled with its good stability and instrument-compatibility, should make EG a promising dye for qPCR and related applications.

## Background

Quantitative PCR (qPCR), also known as real-time PCR, has become a powerful tool for the amplification, identification and quantification of nucleic acids. Its ability to quantitatively and specifically detect genes has been invaluable for both research and diagnostic applications [1]. Key to the detection is a fluorescence reporter molecule that monitors the progress of the DNA amplification. Depending on the mode of signal generation, the reporter molecule may either be a fluorogenically labeled oligonucleotide, commonly referred to as a probe, or a simple fluorogenic DNA-binding dye. The most widely used fluorogenic probes are TaqMan probes, which hybridize to an amplicon during the chain extension phase and become subsequently cleaved by *Taq *polymerase through its 5'-3' exonuclease activity, resulting in a fluorescence signal increase [2,3]. Because probe-based qPCR can simultaneously monitor the progress of the DNA amplification and verify the identity of the amplified target, it provides the high sensitivity and specificity required for demanding applications such as medical diagnosis and prognosis without an extra post-PCR product verification step. However, TaqMan probes are usually expensive and time-consuming to synthesize [4,5]. As an alternative to TaqMan probes, simple DNA-binding dyes have been used for qPCR by detecting the total amount of accumulated DNA during the reaction [6]. A PCR dye is universally applicable to any target sequence and is generally far less expensive than an individually synthesized TaqMan probe. Although qPCR using a DNA dye generally does not provide as high a level of specificity as qPCR using a TaqMan-like probe does, its reliability can be verified semi-qualitatively by analyzing the melt curve of the amplicon following the PCR without opening the PCR tubes [7]. For these reasons, qPCR using a simple DNA dye is a popular choice among academic laboratories for routine PCR experiments.

Since Higuchi first demonstrated the use of ethidium bromide in qPCR [6], several other DNA-binding dyes have been tested for the application, including P2 [8], SYBR Green I (SG) [7], SYTO9 [9], and more recently, BEBO and BOXTO [10]. Among the dyes screened, SG is by far the best performing one and consequently has become the most widely used qPCR dye. SG is highly fluorescent when bound to DNA with very little background fluorescence. The dye has excitation and emission spectra matching well with the optical settings in most of the instruments, and is largely compatible with the chemistry of PCR. Despite its popularity, SG still has several disadvantages. One of the problems is that only a relatively low SG concentration (e.g. < 0.5 μM) can be used in the reaction because of the dye's high tendency to inhibit PCR and promote mispriming [11,12]. Low SG concentration may not only compromise PCR signal strength but also may make DNA melt curve data unreliable due to the so-called "dye redistribution" phenomenon [13]. The latter problem has been cited to be the main reason why SG is unsuitable for high-resolution melt curve analysis (HRM), which is now gaining popularity because of the high specificity it offers and its potential to be used in diagnostic applications. In addition, SG is relatively unstable, especially under the alkaline condition of Tris buffer during storage [11]. Finally, although SG is only weakly mutagenic by itself [14], it has been reported to be a strong mutation enhancer by possibly impairing the natural DNA repair mechanism in cells [15].

EvaGreen (EG) is a new DNA-binding dye we developed. Since its commercialization, it has been reported to be used for DNA quantification [16,17], double-stranded DNA tracing and quantification in capillary electrophoresis [18], DNA conformation detection [19], quantitative PCR [20,21], melting analysis on a Lab-on-Chip [22], and real time isothermal DNA amplifications [23]. In this study, we take a systematic approach to evaluate the new dye in spectral properties, stability, DNA-binding affinities, qPCR and post-PCR DNA melt curve analysis. By changing dye concentration, chain extension time or the length of amplicons respectively while keeping other PCR components constant, we studied how each of the factors affected Ct or specificity in qPCR. We also studied EG's DNA-binding behavior in side by side comparison with SG towards double stranded DNA vs. short stranded DNA, long (>20 bp) vs short (10 bp). Finally, we attempt to use all the physicochemical properties of EG to explain why EG showed significant advantage in qPCR and post-PCR DNA melt curve analysis.

## Results

### Absorption and fluorescence spectra

**Figure 1:**
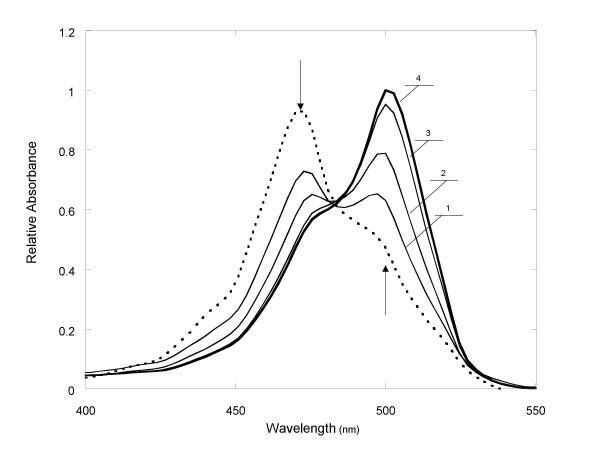
Absorption spectra of EG in the presence of various amounts of λDNA: 0 ng/μL (dotted line); 5 ng/μL (line 1), 10 ng/μL (line 2), 25 ng/μL (line 3) and 100 ng/μL (line 4). All spectra were measured at room temperature in 100 mM Tris buffer, pH 8.0 with an EG concentration of 11.15 μM. The arrows indicate trends of absorbance change in response to the increase of DNA amount. All absorbance points are relative to the maximum of line 4.

**Figure 2:**
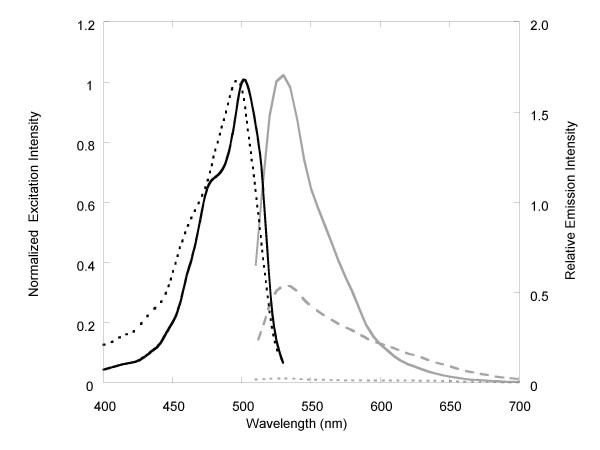
Excitation spectra of EG free dye (dark dotted line) and EG in the presence of λDNA at 40 ng/μL (dark solid line) and emission spectra of EG free dye (gray dotted line), EG in the presence of λDNA at 40 ng/μL (gray solid line), and EG in the presence of M13 DNA at 40 ng/μL (gray dashed line). The excitation spectra were normalized to the same intensity to better compare the relative wavelength shift while the emission spectra were plotted in relative intensities in order to compare the fluorescence brightness of the dye under different conditions. All spectra were measured at room temperature in AmpliTaq DNA polymerase buffer (ABI) with a dye concentration of 11.42 μM.

The absorption spectrum of EG without DNA presence showed a major peak at 470 nm and a shoulder peak at around 495 nm (Figure 1). Upon addition of increasing amounts of dsDNA (λDNA), the peak at 470 nm dropped while a new peak at 500 nm emerged. At saturating DNA concentrations, the 470 nm peak decreased to a shoulder peak while the peak at 500 nm became the major peak. Without DNA present, the dye was weakly fluorescent with an excitation peak at 495 nm and emission peak at 525 nm (Figures 2). In the presence of dsDNA, the excitation and emission wavelengths of the dye red shifted to 500 nm and 530 nm, respectively. By comparing the dye's fluorescence intensities at zero and saturating (i.e., 40 ng/μL) λDNA concentrations, the fluorescence enhancement of the dye upon DNA binding was estimated to be approximately 70 times (Figure 2). In the presence of the same concentration of M13 DNA, which is considered to be mostly single-stranded with some local double-stranded regions, the fluorescence enhancement was only about 21 times.

### DNA titration

**Figure 3:**
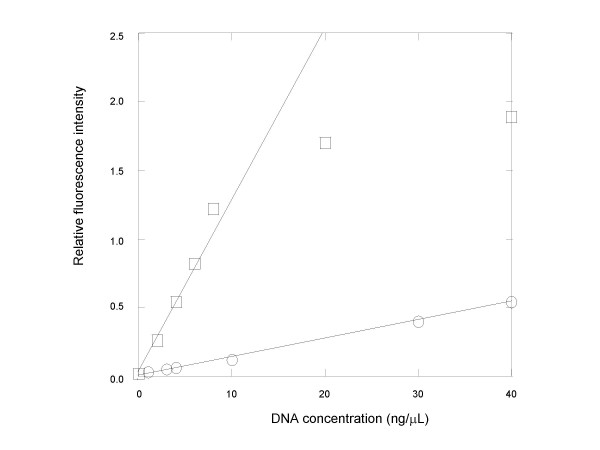
Fluorescence intensity of EG (11.15 μM) in response to titration using single-stranded M13 DNA (circle) and double-stranded λDNA (square), respectively. The linear response range for double stranded DNA titration is from 0 to about 10 ng/μL with a slope of 0.148 while the linear response range for single-stranded DNA titration is from 0 to at least 40 ng/μL with a slope of 0.013. Thus, the ratio of the two slopes is 11.4.

To gain further insight into the fluorescence responses of the dye toward dsDNA and ssDNA over a wider range of DNA concentrations, the dye was titrated with λDNA and M13 DNA, respectively, in 1× AmpliTaq buffer. The fluorescence intensity readings for each titration were plotted against the DNA concentrations as shown in Figure 3. The linear titration range for λDNA was from 0 to about 10 ng/μL with a slope of 0.148 while the linear titration range for M13 DNA was from 0 to at least 40 ng/μL with a slope of 0.013. The ratio of the two slopes is thus 11.4, indicating that within the linear titration range the dye is more than 11 times more fluorescent in the presence of dsDNA than in the presence of ssDNA of the same weight concentration.

**Figure 4:**
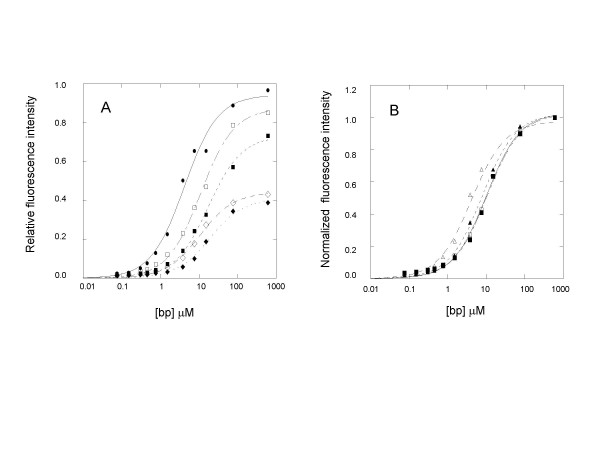
Panel A. Fluorescence of EG (at 11.15 μM) and SG (at 11.42 μM) in response to input DNA in various media at 50°C: EG + λDNA in 100 mM Tris (open squares) or in 10 mM Tris containing 100 mM KCl (open diamonds); EG + Tbr DNA in 100 mM Tris (solid diamonds) or in 10 mM Tris containing 100 mM KCl; EG + Tbr DNA in 100 mM Tris (solid square) or in 10 mM Tris containing 100 mM KCl (solid diamond); and SG + λDNA in 100 mM Tris (solid circle). Panel B: Normalized DNA binding curves. The fluorescence of EG and SG with various DNA in 100 mM Tris pH 8.0 at 50°C was normalized to better compare the relative positions of the binding curves on the X axis. EG + λDNA (open square); EG + Tbr DNA (solid square); SG + λDNA (open triangle); and SG + Tbr DNA (solid triangle). The relative positions of the DNA binding curves show that the affinity of SG for λDNA is higher than that for Tbr DNA, and the affinities of EG for λDNA and Tbr DNA are about equal and are lower than that of SG for either type of DNA.

In order to assess whether EG binds to dsDNA with any base preference and whether its DNA binding is affected by salts, the dye was titrated with the double-stranded AT-rich λDNA and the double-stranded GC-rich Tbr DNA, respectively, in Tris buffers with or without an inorganic salt. Furthermore, in order to mimic the condition used during qPCR, the titrations and fluorescence recordings were made at 50°C. As shown in Figure 4A, the fluorescence of the dye in the presence of λDNA (open square) is twice as intense as that in the presence of Tbr DNA (open diamond) in 100 mM Tris pH 8.0. A similar trend was observed when the titrations were carried out in 10 mM Tris pH 8.0 containing 100 mM KCl (Figure 4A, solid square and solid diamond) except that the titration curves were shifted toward the right relative to their counter parts. The large fluorescence difference observed between titration using λDNA and that using Tbr DNA was apparently not due to difference in the dye's binding affinities toward the two types of DNA. This can be better appreciated by replotting the titration curves with normalized fluorescence intensities. As shown in Figure 4B, the normalized titration curves of EG with λDNA and Tbr DNA in 100 mM Tris pH 8.0 are nearly completely superimposable, suggesting that EG binds to the two types of DNA with equal affinity. Likewise, nearly identical normalized titration curves were obtained for EG titrations with λDNA and Tbr DNA in 10 mM Tris pH 8.0 containing 100 mM KCl except that, again, they are shifted to the right relative to the titration curves obtained in 100 mM Tris buffer pH 8.0 (data not shown). The decrease in the dye's DNA binding affinity due to salt effect, as suggested by the shifting of the DNA binding curves to the right, was also observed when the 100 mM KCl in the buffer was replaced with 100 mM MgCl_2 _(data not shown).

For comparison, SG was also titrated with λDNA and Tbr DNA, respectively, in 100 mM Tris pH 8.0, and the resulting normalized titration curves were co-plotted with those of EG (Figure 4A and 4B). Unlike EG, SG displayed differential binding affinities towards the two types of DNA; the significant left-shifting of the SG titration curve with λDNA suggests that SG has a higher affinity toward λDNA than toward Tbr DNA. Furthermore, as shown in Figure 4B, SG has an overall higher DNA binding affinity than EG does. However, similar to EG, SG also produced nearly 50% less fluorescence when titrated with the GC-rich Tbr DNA, compared to titration with the AT-rich λDNA (data not shown).

**Figure 5:**
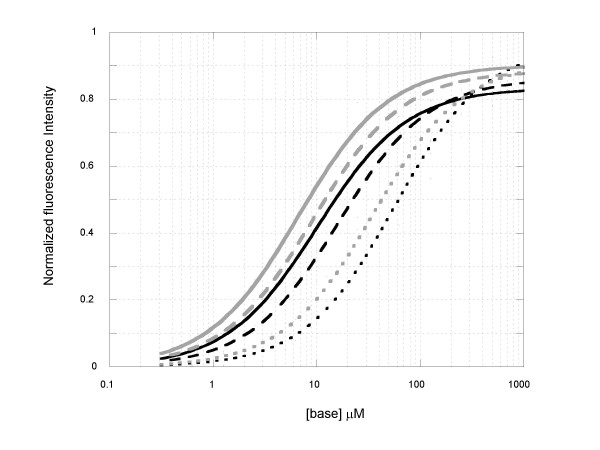
Normalized binding curves of EG (at 11.15 μM) and SG (at 11.42 μM) with various oligonucleotides in 100 mM Tris, pH 8.0 at room temperature : 1) EG + ds22-mer (dark solid line); 2) EG + ds10-mer (dark dashed line); 3) EG + ss24-mer (dark dotted line); 4) SG + ds22-mer (gray solid line); 5) SG + ds10-mer (gray dashed line); and 6) SG + ss24-mer (gray dotted line).

To learn how EG and SG each may bind to primers, primer-dimers and primer-template hybrids in typical PCR reactions, each dye was titrated with a 10-base paired stem-loop fragment (ds10-mer), a 22-base paired stem-loop fragment (ds22-mer), and a 24-base single-stranded fragment (ss24-mer). To ensure the conformation stability of the double-stranded fragments and detection sensitivity, the comparative binding study of the dyes with the three DNA fragments was carried out at room temperature instead of a higher temperature as used in PCR. As shown by the normalized DNA binding curves in Figure 5, both EG and SG exhibited lower binding affinity toward shorter dsDNA fragment (i.e., ds10-mer) than longer dsDNA fragment (i.e., ds22-mer), and even lower affinity toward the ssDNA fragment except that EG generally showed lower affinity than SG for each given type of DNA fragment. As the DNA concentration corresponding to the midlevel fluorescence of a binding curve may be used to estimate the relative dissociation constant of a DNA-dye complex [24], it is evident that the degree of binding affinity decrease for EG as a result of DNA fragment size reduction or change from dsDNA to ssDNA fragment was far more pronounced than that for SG.

### Effect of dye concentration on qPCR performance

**Figure 6:**
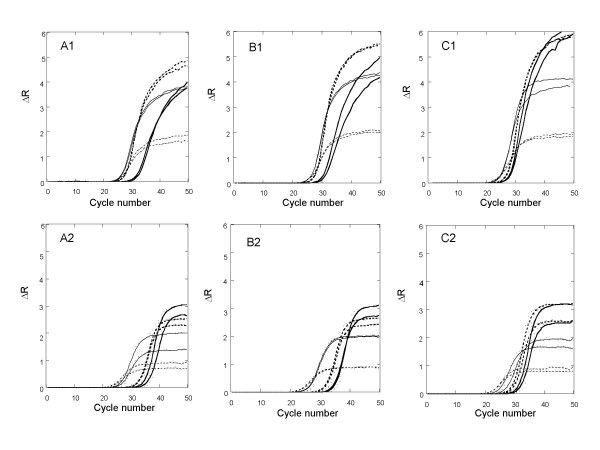
Effect of EG and SG concentrations on PCR performance: Amplification plots of McG (Panels A1, and A2), TBP (Panels B1 and B2) and GCL (Panels C1 and C2) using EG (Panels A1, B1 and C1) or SG (Panel A2, B2 and C2) as the DNA-binding dye for real-time fluorescence monitoring. Each template was amplified in duplicate under four different concentrations of each dye: 2× (thick solid line), 1.5× (thick dashed line), 1× (thin solid line), and 0.5× (thin dashed line) (1 × SG = 1.33 μM; 1 × SG = 0.67 μM). At 1.5 × SG and 2 × SG, amplifications of both McG and TBP produced non-specific products as revealed by post-PCR DNA melt curve analysis and agarose gel electrophoresis.

SYBR Green I is known to show PCR inhibition when used at above a certain threshold concentration [9,11,25,26]. The exact threshold concentration may vary, depending on such factors as the type or make of DNA polymerase, primer sequences, amplicon, buffer components and cycling parameters. It is desirable to have a relatively high threshold dye concentration so that PCR signals can be made more robust and DNA melt curve data more reliable (See discussion section). To explore the concentration limit for EG, we conducted qPCR experiments using four separate dye concentrations, 2.66 μM (2×), 2 μM (1.5×), 1.33 μM (1×) and 0.67 μM (0.5×), respectively. Also, to test the effect of amplicon size, each dye concentration was applied to the amplifications of three separate amplicons, McG (121 bp), TBP (228 bp), and GCL (529 bp), all cloned into a plasmid (Figure 6, Panels A1, B1 and C1). For comparison, similar experiments were carried out using four SG concentrations, 1.36 μM (2×), 1.02 μM (1.5×), 0.68 μM (1×) and 0.34 μM (0.5×), respectively, under the same condition (Figure 6, Panels A2, B2 and C2). The concentrations of SG were determined using a SG extinction coefficient of 58,000 at 494 nm, which we estimated using a combination of the dye's optical density specification supplied by the manufacturer and published information on the dye's structure [27]. This number is significantly lower than the reported extinction coefficient of 73,000 (see detailed explanation in Methods).

To eliminate any differences due to primer annealing, the universal M13 forward and reverse primers were used for all PCR experiments. Also, the denaturing and annealing steps in each thermo cycle were each set for 15 seconds to ensure that the two processes were as close to completion as possible in all experiments.

Using the standard PCR protocol (60 seconds at 72°C), EG at 0.5× (i.e., 0.67 μM) and 1× both gave relatively early Ct for all three amplicons. Slight Ct delays occurred at 1.5 × EG while raising the dye concentration to 2× caused significant Ct delays (Figure 6, Panels A1, B1, and C1). For SG, the only non-inhibitory concentration was 0.5× (i.e., 0.34 μM) for all three amplicons (Figure 6, Panels A2, B2 and C2). Higher SG concentrations resulted in either significant Ct delay or non-specific amplifications as revealed by melt curve analysis and gel electrophoresis (data not shown). Thus, based on these results, the threshold concentrations (i.e., the highest possible dye concentrations without causing significant Ct delay and nonspecific product formation) under our conditions are approximately 1.33 μM for EG and 0.34 μM for SG, respectively. As shown in Figure 6, qPCR using EG on average gave 4–5 times more fluorescence than that using SG when both dyes were applied at their threshold concentrations. It is also worth noting that the effect of target sequence on Ct differed slightly between reactions using the two dyes. For example, for the amplifications of McG and GCL, EG gave earlier Ct values than SG. On the other hand, for the amplification of TBP, SG gave earlier Ct values than EG. In general, however, the Ct difference between two dyes for a given amplicon was within ± 1 cycle.

### Effect of chain extension time and amplicon size on qPCR performance

Given the finding that EG had relatively low PCR inhibition, we reasoned that it might be possible to reduce the chain extension time from the standard 60 seconds to a shorter time without adversely affecting PCR performance. Thus, we compared EG and SG in qPCR experiments using the extension time at 72°C of 30, 15 and 5 seconds, respectively. In order to see the interplay of dye concentration and chain extension time, each dye was also tested at two different concentrations, i.e., 0.67 μM (0.5×) and 1.33 μM (1×) for EG and 0.34 μM (0.5×) and 0.68 μM (1×) for SG, respectively. Again, McG, TBP and GCL were chosen to represent amplicons of different sizes in the comparative studies.

**Figure 7:**
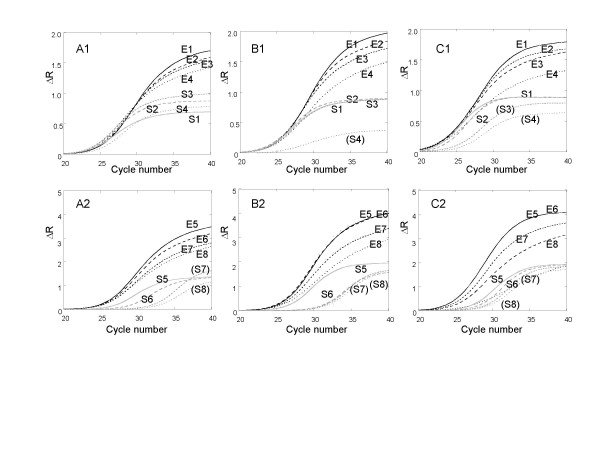
Effect of chain extension time on qPCR using EG or SG: Amplification plots of McG (Panel A1, and A2), TBP (Panel B1 and B2) and GCL (Panel C1 and C2) with EG or SG at 0.5× or 1× dye concentration: 0.5 × EG (dark lines E1–E4 in panels A1, B1 and C1); 1 × EG (dark lines E5–E8 in panels A2, B2 and C2); 0.5 × SG (gray lines S1–S4 in panels A1, B1 and C1); 1 × SG (gray lines S5–S8 in panels A2, B2 and C2). Four different elongation times, *i.e., *60 seconds (lines E1, E5, S1 and S5), 30 seconds (lines E2, E6, S2 and S6), 15 seconds (lines E3, E7, S3 and S7) and 5 seconds (lines E4, E8, S4 and S8), were used for the amplification of each gene fragment with each dye and dye concentration. Line trace numbers within parenthesis indicate formation of nonspecific products as confirmed by post PCR DNA melt curve analysis.

As shown in Figure 7, panel A1, reduction of chain extension time did not significantly affect the Ct values for McG amplifications for either dye at 0.5× concentration although amplifications with EG consistently gave about twice the final fluorescence signal as those with SG did. Doubling the concentration of EG from 0.5× to 1× had no major effect on the amplification in terms of both Ct values and specificity (Figure 7, panel A2). In contrast, when the concentration of SG was doubled to 1×, the Ct became increasingly delayed as the chain extension time was systematically shortened. When the extension time was shortened to 15 seconds or lower, there were marked Ct delays as well as formation of nonspecifically amplified products (Figure 7, panel A2, lines S7 and S8) as confirmed by post-run DNA melt curve analysis (data not shown).

Similar results were obtained in the comparison of EG and SG in the amplifications of the longer 228 base pair TBP and 529 base pair GCL, respectively, except that the Ct delay for the amplifications using SG became even more pronounced as the chain extension time was shortened. Amplification reactions using EG showed significant Ct delay, only under the most challenging condition–the combination of a very long amplicon (529 bp), a relatively high dye concentration (1× or 1.33 μM) and a very short extension time (5 s) (Figure 7, panel C2, line E8). On the other hand, the Ct values for amplification reactions using SG became quite sensitive to reduced extension time, especially at the higher 1× (0.68 μM) dye concentration (Figure 7, panels B2 and C2). Moreover, when the extension time was shortened to 30 seconds or below, amplifications of both TBP and GCL using SG produced nonspecific products (Figure 7, panels B2 and C2, lines S7 and S8). In fact, when the extension time was reduced to 5 seconds, only nonspecifically amplified products were detected as revealed by post-PCR DNA melt curve analysis and gel electrophoresis (data not shown). Finally, reactions using SG generally showed poorer reproducibility than those using EG in response to decreasing extension time and elevated dye concentration (data not shown).

**Figure 8:**
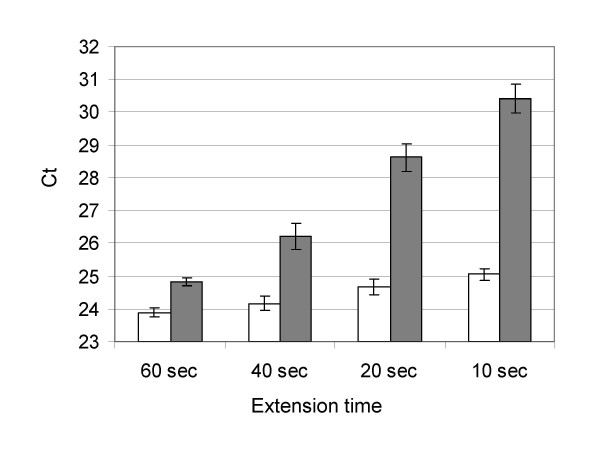
Effect of chain extension time reduction on the Ct values and PCR reproducibility of EG master mix and Power SYBR master mix (ABI). GAPDH was amplified with an EG master mix or Power SYBR master mix using the universal protocol with 15 seconds at 95°C and various chain extension times (60, 40, 20 and 10 seconds, respectively) at 60°C. The two-temperature cycling protocol was recommended by the manufacture (Applied Biosystems) for its Power SYBR master mix. Each amplification was repeated 8 times in order to assess the reproducibility. As the chain extension time was shortened from 60 seconds to 10 seconds, the average Ct value for the reactions using EG master mix increased by about 1 cycle while the standard deviation among octuplicates increased slightly from 0.14 cycle number in 60-second extension time to 0.23 cycle number in 20-second extension time (open bars). Under similar conditions, the average Ct for the amplification using Power SYBR (shaded bars) increased by more than 5 cycles and the standard deviation for Ct increased from 0.10 to 0.43 cycle.

The above results suggest that EG may be suitable for fast cycling qPCR. Thus, to further explore this possibility, we carried out qPCR on a GAPDH fragment using the optimal 1.33 μM EG concentration. The reactions were performed using two-temperature cycling protocols of 15 seconds at 95°C and various amounts of reduced annealing/extension time (i.e., 60, 40, 20 and 10 seconds, respectively) at 60°C. For comparison, a commercially available kit, Power SYBR Master Mix, was run side-by-side, also using the same two-temperature cycling protocols. The commercial master mix contained approximately ~0.3 μM SG as determined from the dye's optical density at 494 nm (See Method section for SG concentration determination). Instead of making our own SG master mix from the same buffer and enzyme system used for EG, we chose Power SYBR Master Mix to avoid the possibility that the our buffer and enzyme system might show bias against SG and because Power SYBR Master Mix was of optimized formulation for SG as suggested by the manufacturer. Each amplification reaction was repeated eight times in order to assess the reproducibility of the reaction. Figure 8 displays the Ct values of amplification reactions run with various reduced annealing/extension times for Power SYBR Master Mix and the EG master mix. When the annealing/extension time was set for 60 seconds, amplifications with either master mix gave comparable Ct values and similar reproducibility. However, as the annealing/extension time was shortened, the Ct for Power SYBR Master Mix became increasingly delayed and its reproducibility, as shown by the error bars in the figure, also suffered progressively. For example, the Ct for Power SYBR was delayed by as many as 5–6 cycles when the annealing/extension time was reduced to 10 s. On the other hand, for the same extent of annealing/extension time reduction, the Ct for the EG master mix increased by only about 1 cycle while still maintaining excellent reproducibility.

### PCR dynamic range

**Figure 9:**
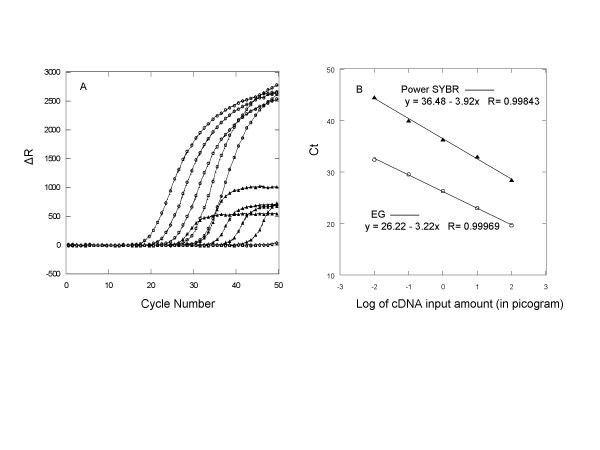
Amplification plots (Panel A) of GAPDH from various input of cDNA using either EG (open circle) or SG (solid triangle) as the DNA-binding dye in the PCR mix. All reactions were run in duplicates, but only one plot per duplicate is shown for clarity. Plots of Ct value vs. logarithm cDNA input amount for both sets of reactions are shown in panel B. Reactions with EG (open circle) showed earlier Ct values and high amplification efficiency.

A key application of qPCR is to determine the starting concentration of a target gene. This is typically accomplished by comparing the Ct for the target gene amplification of the sample against a standard qPCR titration curve, where the Ct is linearly related to the logarithm of the starting DNA copy number. Among other factors, the linear dynamic range of the PCR titration is expected to be affected by the qPCR dye. Thus, to compare EG and SG for their performance in this aspect of qPCR, 10-fold serial dilutions of a GAPDH target as a cDNA preparation were amplified using either an EG master mix or Power SYBR master mix. Following each PCR experiment, DNA melt curve analysis was carried out to verify amplification specificity. As shown in Figure 9, target amplifications in the presence of either dye produced comparably good linear dynamic range, efficiency and specificity. However, amplifications using the EG mix resulted in significantly earlier Ct values and higher signals than those using Power SYBR mix. In addition, the fluorescence signals continued to rise for more than 30 cycles for amplifications using the EG mix. On the other hand, for amplifications using Power SYBR mix, the signal increase lasted for only about 10 cycles before reaching a plateau.

### DNA melt curve analysis

**Figure 10:**
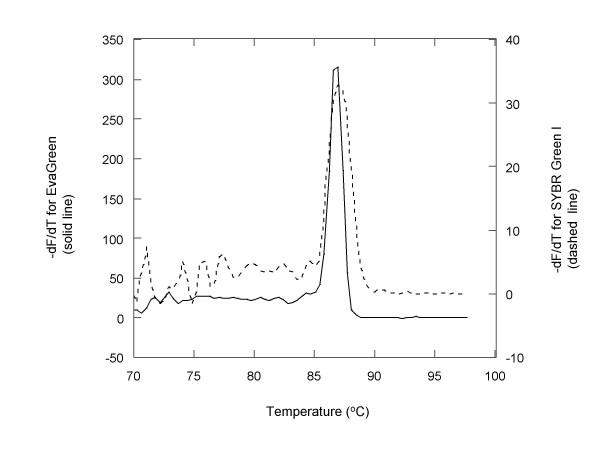
Melt curves of the GAPDH amplicon in the presence of EG (solid line) or SG (dashed line) following the amplification of 100 pg human cDNA input as described for Figure 9. The melt peak recorded with EG is about 10 times more intense than that recorded with SG while its width is only about one half of the latter at the half-peak height.

We observed that DNA melt peaks associated with EG at its optimal concentration (i.e., 1.33 μM) were significantly stronger and also narrower than those associated with SG at its optimal concentration. In addition, the melt temperatures for DNA associated with EG were generally from 0.5 to about 1°C lower than those for DNA associated with SG in the same buffer. Figure 10 compares the melt curves of two GAPDH amplicons, each resulting from the amplification of 100 pg starting human cDNA using either a EG mix or Power SYBR mix as described above for the human cDNA titration experiments. As shown in the figure, the peak width at half height for EG (~1.2°C) is only about half of that for SG (~2.2°C) while the peak height for the former is nearly ten times that for the latter. In order to rule out the possibility that the apparently wider peak associated with SG was due to multiple peaks superimposed with each other, we examined the products from both PCR reactions on agarose gels. The results indicated that the PCR products from both reactions were the same single product.

### Effect of DNA polymerase source on the comparative performance of EG and SG in qPCR

The qPCR data shown in Figure 6 and 7 were all generated with *AmpliTaq *from ABI. As DNA polymerases from different commercial sources may be prepared differently and supplied in different storage buffers, we would like to find out if the make of enzyme played any role in the observed qPCR performance difference between EG and SG. The experiments were repeated with Taq from NEB, Promega, Fermenta and home made Taq respectively. We found that although Ct values and PCR signal strength differed slightly from enzyme to enzyme and from run to run, the performance difference between the two dyes still held (data not shown).

### Stability of EvaGreen

**Figure 11:**
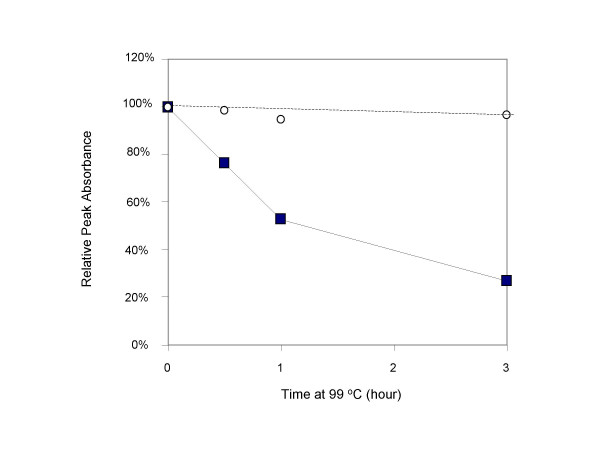
Stability comparison between EvaGreen (open circle) and SYBR Green I (filled square). EvaGreen (11.42 μM) and SYBR Green I (12.83 μM) were each incubated in 100 mM Tris pH 8.0 at 99°C and sampled at time 0, 0.5, 1 and 3 hours for absorption spectrum measurement at room temperature. ROX was added to each solution to give an absorbance of 0.17 at 580 nm and was used as a stable reference to correct for spectral baseline shift due to either solvent evaporation or instrument instability. The normalized peak intensities of each dye in absorbance at each time points (relative to 0 hour) were plotted against the hours the dye stayed at 99°C.

qPCR exposes reaction components to high temperature and, in some PCR instruments, to high powered laser irradiation. Moreover, the pH of Tris-based master mixes is often alkaline at room temperature and especially at 4°C during storage. Thus, it is essential for a qPCR dye to be thermally, hydrolytically and photolytically stable. To test the thermal and hydrolytical stability of EG under an accelerated condition, a solution of the dye along with a reference dye ROX was incubated in pH 8.0 Tris at 99°C for three hours. During the course of incubation, the solution was periodically sampled for absorption spectral measurement at room temperature. For comparison purpose, a solution of SG and ROX reference dye in the same buffer was incubated and monitored under the same condition. As shown in Figure 11, the absorption spectrum of EG shifted only slightly, a result largely within the experimental error. In contrast, the spectrum of SG had nearly disappeared at the end of the 3-hour incubation. Similar data were obtained when the dyes were incubated in pH 9.0 Tris buffer at the same temperature except that SG decomposed even faster (data not shown). To test the ability of EG to withstand multiple freeze-and-thaw cycles, a 26.7 μM EG solution in H_2_O (EvaGreen 20× from Biotium, Inc.) was subject to 10 freeze-and-thaw cycles by taking the sample in and out of a -20°C freezer over the course of one week. UV/Vis measurements of the EG sample before and after the freeze-thaw test detected no significant change (data not shown). EG also appeared to be reasonably photostable as no signal variation was observed during routine qPCR experiments due to photobleaching of the dye.

## Discussion

QPCR using SG has proven to be a valuable alternative to that using the more costly TaqMan probes, particularly for many routine research applications. However, problems with SG's tendency to inhibit PCR and promote nonspecific amplification have prevented wider applications of the technique [28]. Furthermore, the low dye concentration required to minimize SG's interference to PCR can cause dye redistribution problem during DNA melt curve analysis, making the dye unsuitable for repeat melting, multiplex PCR [29] and high-resolution genotyping [30]. We have demonstrated that EG is a promising new DNA-binding dye with several features that make the dye suitable for both qPCR and challenging post-PCR melt curve analyses.

For qPCR employing a DNA-binding dye, an inherently conflicting issue is how much dye should be used in order to achieve the best result possible. On the one hand, a relatively high dye concentration should result in a high fluorescence signal in response to the increasing amount of DNA generated during the reaction. Although the Ct value of the reaction is not determined by the fluorescence signal strength, an overall higher signal level for the amplification adds robustness to the qPCR by increasing the confidence level of the fluorescence readings. Moreover, a relatively high end-point fluorescence signal should directly result in a relatively high DNA melt peak during post-PCR melt curve analysis (*vide infra*). On the other hand, because the dye is a DNA-binding molecule, it will inevitably affect the DNA amplification process if present at too high a concentration. Consequently, there exists an optimal dye concentration that gives the best possible fluorescence signal and at the same time ensures minimal or no interference from the dye. We have determined that, under the condition we used, the best concentrations for EG and SG are 1.33 μM and 0.34 μM, respectively. The significantly higher concentration permitted for EG suggests that EG has much lower PCR inhibition than SG does. With the respective optimal dye concentrations, qPCR using EG consistently yields 4 to 5 times more fluorescence than that using SG does, leading to overall more robust results (Figures 6, 7, and 9).

The relatively low PCR interference but high PCR signal of EG may have stemmed largely from its favorable DNA binding properties. The PCR inhibition by a dye, as characterized by a delay in Ct, is most likely due to the dye's interference with the chain extension step, where the dye may bind to the single-stranded template, thereby serving as "road bumps" to the extending primer-template complex. Therefore, an ideal qPCR dye should preferentially bind to dsDNA and have as little interaction as possible with ssDNA. As shown in Figure 5, EG has much lower affinity towards ssDNA than SG does. This DNA binding difference between the two dyes may be at least partially responsible for their difference in the PCR inhibition. The relatively high PCR inhibition we observed for SG is also consistent with data reported for other similar dyes that have high affinity for single stranded oligonucleotides. It is known that multi-cationically charged nucleic acid dyes such as TOTO-1, ethidium homodimer-1, PicoGreen and TO-PRO are generally not good dyes for qPCR [[8], and unpublished observations]. These highly positively charged dyes typically exhibit poor binding selectivity between dsDNA and ssDNA because a large portion of their DNA binding affinity is attributable to electrostatic interaction, for which ssDNA is also effective. Another possible contributing factor to PCR inhibition is the high binding affinity of the dye to dsDNA, particularly to short dsDNA fragments. During the chain extension phase, portion(s) of the single stranded template may form regional stem-loop structure(s), which may be transient and unstable in nature but could be stabilized in the presence of a high affinity dsDNA-binding dye. It would not be surprising that the stabilization of such secondary structures could lead to a significant slow down of the polymerization reaction, particularly for long amplicons, where stem-loop secondary structures are more likely to form. As demonstrated in Figures 4 and 5, the binding affinities of both EG and SG become progressively lower as the size of dsDNA becomes smaller. However, the extent by which the binding affinity is weakened is much more significant for EG than for SG. The weaker affinity of EG toward short dsDNA fragments should be of advantage because the dye is less likely to stabilize any regional stem-loop structures in the template and thus less likely to inhibit PCR via this mode of PCR interference.

The weak binding of EG toward short dsDNA fragments may also help explain the dye's relatively low tendency to promote nonspecific amplifications. Nonspecifically amplified products are a result of mis-priming, where unintended chain extension reactions are initiated either by the set of primers partially hybridizing to each other or by the primers partially hybridizing to unintended regions of the templates. These PCR side-products can sometimes become the dominant or even the exclusive products under certain challenging conditions such as high dye concentrations, long amplicons, low target concentrations and the use of a non-hot-start enzyme, for example. The highly dye-dependent and dye concentration-dependent nature of nonspecific product formation strongly suggest that certain DNA-binding dyes such as SG may have the ability to enhance or promote the formation of nonspecific primer hybridizations, which would otherwise not occur due to imperfect sequence match. Indeed, intercalating dyes are known to be able to increase the stability of DNA or RNA duplexes by enhancing the stacking interaction and reducing the charge density of the phosphate backbone [31,32]. Because the duplexes formed from mis-priming are very short fragments, the binding affinity of a qPCR dye to short dsDNA fragments should correlate well with the dye's tendency to promote nonspecific amplifications. Figures 6 and 7 show that, compared with SG, EG is much less likely to cause nonspecific amplification, consistent with EG's lower binding affinity to short dsDNA fragments. One should realize that a more accurate assessment on EG's lower tendency to cause nonspecific amplification will likely require more rigorous testing methods such as cloning and sequencing of a large number of different clones.

Fast qPCR is attractive for the amount of time one could save. However, qPCR using SG has been reported to produce poor reproducibility [33], which has been confirmed by our result (Figures 7 and 8). We have demonstrated that, as a result of EG's lower tendency to inhibit PCR and promote nonspecific amplification, qPCR using the dye could be reliably carried out with up to 1.33 μM dye concentration and as little as 10 s of chain extension time (Figures 6, 7, 8). However, because we used relatively simple plasmid DNA as the input template, one should exercise caution when applying the same conditions to the amplifications of more challenging templates such as genomic DNA. One of the factors that make genomic DNA challenging templates is their resilience to melting in the initial cycles. Also, the large remaining portion of the DNA other than the target region may assume regional conformations that can act as inhibitors for the polymerase, effectively reducing the concentration of the enzyme. In addition, portions of the non-target region of the genomic DNA may form partial or imperfect hybrids with the primers and/or the target and thus may initiate nonspecific amplifications and interfere with the desired target amplification. Furthermore, because of the large size, genomic DNA may significantly increase the viscosity of the reaction solution, thus lowering the diffusion rate of the reacting molecules. In view of these factors and the dye effect, several steps may be taken to ensure the success of amplifying a genomic target: (a) adjust the buffer composition or add an organic solvent such as DMSO [34] to facilitate the DNA melting; (b) extend the melting time; (c) avoid excessive reduction of the extension time; (d) lower the dye concentration; and (e) increase the amount of enzyme.

PCR buffer composition may play a role in the performance of an EG master mix. It should be emphasized that all qPCR tests described in this paper employed regular *Taq *and were run in a buffer containing 10 mM Tris pH 8.3, 50 mM KCl and 2.5 mM MgCl_2_, as recommended by the enzyme manufacturer. For qPCR using chemically-modified hot-start enzyme AmpliTaq Gold, a cognate buffer containing 10 mM Tris pH 8.0, 50 mM KCl and 2.5 mM MgCl_2 _is usually recommended by the enzyme suppliers. Although these buffers have been widely used for qPCR using SG, our preliminary results indicate that they may not be optimal for qPCR using EG in combination with chemically-modified hot-start *Taq*. EG may perform even better with a lower KCl concentration and concurrently a higher Tris concentration, particularly for high-GC amplicons (data not shown). The difference in buffer preference between EG and SG may be a reflection of the different DNA-binding profiles of the two dyes, and suggests that for different enzymes it may be necessary to adjust the buffer composition in order to best take the advantage of EG.

Our data showed that EG gave much stronger and narrower DNA melt peaks than SG did in post-PCR DNA melt curve analysis (Figure 10), a techniques increasingly used as a better alternative to gel electrophoresis for PCR product confirmation [7]. Two factors may have contributed to the substantial difference in the melt peak intensities associated with the two dyes. The primary factor was likely to be the difference in dye concentrations used in the qPCR experiments. Because a relatively high EG concentration (i.e., 1.33 μM) was used in the qPCR, the fluorescence resulting from dsDNA-dye complex formation at the end of the amplification reaction was accordingly high. The high starting fluorescence level led to a large fluorescence fall following dsDNA melting. As the intensity of a DNA melt peak measures the rate of fluorescence loss as a function of temperature, a large fluorescence fall corresponds to an intense peak. The second factor was likely to be the difference in the discriminative fluorescence response on bindings to ssDNA and dsDNA between the two dyes. Because EG had greater preferential fluorescence response to dsDNA over ssDNA than SG did (Figure 3), the conversion of a dsDNA-dye complex into a ssDNA-dye complex was expected to result in a greater fluorescence change for EG than for SG. Moreover, EG's significantly lower binding affinity to ssDNA should further decrease the fluorescence following the dsDNA-EG complex melting. Thus, these factors in combination should make post qPCR DNA melt curve analysis using EG much more sensitive than that using SG.

The relatively narrow DNA melt peaks associated with EG may be a result of high dye concentration, which minimizes or prevents dye redistribution problem from occurring. When the amount of a DNA-binding dye is limiting relative to the amount of the amplicon, only a small fraction of the binding sites available on the amplicon may be occupied by the dye molecules. During amplicon melting, the dye molecules in the AT-rich regions are expected to dissociate first due to the relatively lower melt temperatures of the regions. The dissociated dye molecules could then become available for binding to the still un-melted GC-rich regions. This dye redistribution may have the effect of delaying the rate of fluorescence loss during amplicon melting, leading to a relatively broad DNA melting transition and thus broad melt peak. The relatively high PCR inhibition of SG requires that the dye be used at a relatively low concentration (i.e., 0.34 μM or blow), which causes dye redistribution problem during DNA melt curve analysis. As a result, melt peaks of dsDNA-SG complexes are often relatively broad. Dye redistribution is also believed to be responsible for SG's failure in the application of closed-tube high-resolution melt curve analysis (HRM), which is becoming a promising technique for identifying variants of heterozygous and homozygous sequences [30,35]. SG could be used for HRM application, only if the dye is reintroduced at a relatively high concentration at the end of a PCR experiment [36]. This practice, however, is undesirable because it is not compatible with qPCR and prone to cross contamination. Unlike SG, EG is compatible with both qPCR and closed-tube HRM [37-39], as a result of the dye's relatively high concentration permitted in qPCR. Therefore the higher permissible concentration of EG in qPCR makes it more advantageous in detecting multiple products by melt curve analysis following the multiplex PCR experiments. It was shown that SG failed to detect multiple PCR products by showing only a single melt peak even though gel electrophoresis revealed the presence of multiple products [36]. The problem was again attributed to insufficient dye presence, which led to SG migration from the low-melting amplicons to the highest-melting amplicon during the DNA melting process.

In a HRM study of methylation differences in an amplicon containing 21 CpG dinucleotides at the SNPRN locus, EvaGreen was compared with LCGreen Plus and Syto9 in statistically significant number of samples. EvaGreen gave most robust result and lowest failure rate [37].

We have shown that EG also possesses other physical properties that should make the dye readily adaptable to current instrument settings and protocols for both qPCR and melt curve analysis. The absorption or excitation peak at 500 nm of the DNA-bound EG well encompasses the wavelengths of some of the common excitation sources such as the 488 nm argon laser or the blue LED light equipped in commercial qPCR instruments. The emission wavelength (530 nm) of the DNA-bound dye also matches well with the FAM detection channel. In the absence of DNA, EG exhibits a weak background fluorescence, which is slightly more pronounced than SG. This turned out to be a benefit for the Bio-Rad iCycler IQ PCR instrument because the background fluorescence of EG is sufficient for well-factor correction, which is normally accomplished by adding FAM to the PCR mix. The nucleotide-independent DNA binding property of EG also could be an advantage, particularly for multiplex PCR, where multiple templates may be amplified in a manner more reflective of the individual template concentrations. We observed that EG appears to be stable both under typical PCR condition and during storage in an alkaline buffer at lower temperatures, thus facilitating its routine handlings.

## Conclusion

Our results suggest that the unique DNA-binding properties of EG could be responsible for the observed relatively low PCR inhibition and relatively low tendency to cause nonspecific amplification by the dye. An ideal qPCR dye is supposed to possess optimal affinity for dsDNA to ensure detection sensitivity without adversely affecting the dsDNA melting step or promoting regional stem-loop structure during the chain extension phase, and at the same time it should have low or no affinity for ssDNA and short DNA fragments. We therefore propose that a DNA dye that substantially relies on electrostatic interaction for its DNA binding may not be ideal for qPCR application because such a dye may bind to ssDNA with significant affinity and is thus more likely to interfere with the chain extension step. The relatively low PCR inhibition of EG can be exploited for several benefits. First, the dye may be used at a relatively high concentration (i.e., 1.34 μM), which should provide a robust PCR signal as well as a strong and sharp DNA melt peak. Second, the use of a relatively high dye concentration in qPCR eliminates dye redistribution problem, making the dye suitable for HRM application in a closed-tube format. Third, EG may be suitable for qPCR using a fast cycling protocol. Finally, we have also shown that EG is both spectrally compatible with existing instruments and very stable. These combined features of EG should make it a promising dye for both qPCR and other related applications.

## Methods

### Materials

EvaGreen (EG) at 20× was from Biotium (Hayward, CA). SYBR Green I (SG) at 10,000× in DMSO was from Molecular Probes (Eugene, OR). *Lambda *DNA (λDNA), *Thermus brokianus *genomic DNA (Tbr DNA) and cDNA were from New England Biolabs (Ipswich, MA), ATCC (Manassas, VA) and Clontech (Mountain View, CA), respectively. All oligonucleotides were synthesized on an Experdite 8909 oligonucleotide synthesizer (Applied Biosystems, Inc., Foster City, CA). Power SYBR Master Mix was from Applied Biosystems, Inc. Other common chemicals were purchased from VWR.

### Extinction coefficient of SYBR Green I

Zipper, et al. elegantly revealed the structure of SYBR Green I using a combination of analytical techniques [27]. The dye was reported to be an asymmetric cyanine dye with a tertiary amine side-chain. The authors reported an extinction coefficient of 73,000 for the dye, but did not disclose what parameters were used to derive this number. Separately, by using ion chromatography, the authors found the total anion concentration to be at least 18.5 mM. Assuming that total anion concentration equals to SG concentration and "simplicity for manufacturing reasons", they proposed the commercial SG at 10,000× in DMSO to have a molar concentration of ~19.6 mM.

However, we believe that this proposed dye concentration was in error, for that inconsistencies will arise if the number is followed. The manufacturer's certificates of analysis accompanying the dye usually list a lot-specific optical density of 0.48 ± 0.3 at 494 nm following a 1:1205 dilution (which we confirmed experimentally for a specific lot we have). Using 0.48 × 1205 as the average optical density value and the 19.6 mM dye concentration reported by Zipper, et al., the extinction coefficient would have been ~29,500, a number which is not in agreement with the 73,000 M^-1^cm^-1^ value reported by Zipper, et al. and dramatically different from the extinction coefficients of dyes in the same class. For example, TO-PRO has an extinction of about 60,000 (Haugland, R.P. (2001) Handbook of Fluorescent Probes and Research Chemicals, 8th ed. Molecular Probes, Inc. Eugene, OR).

We believe the error originated from the assumption that Zipper et. al. made. They mistakenly treated the tertiary amine side-chain as neutral instead of being protonated in the commercial form. With tertiary amine side-chain and permanently charged tertiary amine in the ring, SG should be dicationic, not monocationic. Dicationic SG and monocationic SG will give the same NMR and MS-FAB results. But, the actual molar concentration of the dye should be only half of the total anion concentration. Based on the ~18.5 mM anion concentration from the Zipper paper, the dye concentration is therefore about ~9.3 mM. For the same reason Zipper gave "for manufacturing reasons", the molar concentration of the 10,000× dye is more likely to be 10 mM. Accordingly, using this dye concentration and the 0.48 × 1205 optical density, we estimate the extinction coefficient to be ~58,000.,

### Spectral measurements

All absorption spectra were measured on a Beckman DU 65i spectrophotometer. Fluorescence spectra were measured on a Jasco F-759 spectrofluorometer. Excitation spectra were recorded by monitoring the emission at 530 nm and emission spectra were collected by exciting at 460 nm. All spectral measurements were taken after a 30-minute incubation.

### DNA titration

DNA titrations of EG and SG were performed on a Spectra Max Gemini XS fluorescence microplate reader (Molecular Devices, Mountain View, CA). The total volume of the solution in each microplate well was kept at 200 μL. Following the mixings of DNA and the dyes, the resulting solutions were incubated for 30 minutes at either room temperature or 50°C before measurements were taken at the same respective incubation temperature. Fluorescence signals were recorded using the optimal excitation/emission wavelengths of EG (490 nm/530 nm) or SG (490 nm/525 nm). Several types of DNA were used in the titration experiments in order to assess the DNA-binding behavior of the dyes. λDNA and Tbr DNA were chosen to represent AT-rich and GC-rich dsDNA, respectively. Two short oligonucleotides each with an intramolecular complementary sequence were synthesized to form a double-stranded 10-mer (ds10-mer, GCGATCACCGTTTCGGTGATCGC) and a double-stranded 22-mer (ds22-mer, GCGATCACCGTAATGCTAGCGCTTTTGCGCTAGCATTACGGTAGATCGC), respectively. To ensure the proper formation of the double-stranded structures, stock solutions of the oligonucleotides in 10 mM Tris pH 8.0 were heated to 90°C for 10 minutes and then quickly snap-cooled in ice-water. M13 DNA was chosen to represent ssDNA. Finally, a 24-base oligonucleotide (ss24-mer, GTCTGATTCATCTGCGTG-CTGACT) was synthesized to represent short single-stranded oligonucleotides.

### Real-time PCR and post-PCR analysis

Unless otherwise stated, typical qPCR experiments were carried out in 20 μL-sized reactions containing 10 mM Tris (pH 8.3), 50 mM KCl, 2.5 mM MgCl_2_, 0.25 mM each of dNTP, 500 nM each of forward and reverse primers, a DNA plasmid template (roughly 10^6 ^copies), one unit of *AmpliTaq *DNA polymerase and EG or SG of a concentration as indicated. Reactions were generally assembled on a 96-well PCR plate on ice. The plate was then immediately placed in an iCycler IQ (BioRad) with the heating block heated above 60°C to ensure hot starting. To test the effect of dye concentration, the standard protocol (i.e., 95°C for 3 minutes followed by 45 cycles of 15 seconds at 95°C, 15 seconds at 50°C, and 60 s at 72°C) was used. The extension time at 72°C of the above protocol was shortened by various amounts as indicated to see how EG or SG affects the kinetics and specificity of the amplification reactions. Power SYBR kit from Applied Biosystems, Inc. and a home-made EG master mix were compared using two-temperature fast protocols with 10 minutes at 95°C followed by 50 cycles of 15 seconds at 95°C and a varying amount of annealing/chain extension time (i.e., 10, 20 and 40 seconds, respectively) at 60°C. PCR titrations of cDNA were carried out using Universal PCR protocol (i.e., 95°C for 10 minutes followed by 50 cycles of 95°C for 15 seconds and 60°C for 60 seconds).

All PCR experiments were run in multiplicates, followed by post-PCR product analysis to confirm product specificity. For the comparative study of Power SYBR kit and EG master mix using fast cycling protocols, each PCR experiment was repeated 8 times. All other PCR experiments were run in duplicates but with only one of the amplification curves shown for the sake of simplicity. For all PCR experiments, post-PCR DNA melt curve analysis was performed to assess amplification specificity. DNA melting was carried out using a temperature ramping rate of 0.2°C per step with a 30-second rest at each step. In case of ambiguity, DNA gel electrophoresis was performed.

To test if different enzymes may affect the relative performance of EG and SG differently, the above experiments (except for the comparison experiment with Power SYBR kit) were repeated with Taq from NEB (Catalog # M0273S), Taq from Promega (Catalog # M1661), Taq from Fermentas(Catalog # EP0401) and home-made Taq.

### Templates and primer sequences

Information on vector, insert sequence, amplicon sizes (inclusive of the M13 forward (5'-CTGCAGGATATCTGGATCCAC-3') and M13 reverse (5'-AACAGCTATGACCATG-3') primers) are listed in Table 1.

**Table 1:** Plasmid templates used in qPCR

**Plasmid Name**	**Parental Vector**	**Amplicon Size**	**Insert Origin**	**5'-End Sequence of the Insert**	**3'-End Sequence of the Insert**
pMcG	pUC18	121	Human myc gene	TCAAGAGGTGCCACGTCTCC	CTGATCTGTCTCAGGACTCT
pTBP	pCRII-TOPO	228	Human TBP cDNA	AGCAAGAAAATATGCTAGAG	TTTCCAGAAACAAAAATAAG
pGCL	pBluescript	529	T5 DNA polymerase	CCAGAACCATACAGAATACC	CCAGAACCATACAGAATACC

The comparative study between Power SYBR master mix and EvaGreen master mix was carried out by amplifying plasmid containing the full-length GAPDH cDNA (pGAPDH) using GAPDH forward primer (GAAGGTGAAGGTCGGAGTC) and GAPDH reverse primer (GAAGATGGTGATGGGATTTTC) (both published in ABI User's Bulletin #2). The PCR titration of human cDNA was carried out using the same set of GAPDH primers as used for the above Power SYBR and EvaGreen comparative study.

### Stability study of EvaGreen and SYBR Green I

The stability study of EvaGreen and SYBR Green I was carried out by comparing the absorption spectra of the dyes in Tris buffer following incubation at 99°C for different amounts of time. One milliliter solution of EG (11.42 μM) or SG (12.83 μM) in 100 mM Tris (pH 9.0 at room temperature) containing ROX (OD_580_nm ≈ 0.17) was prepared. ROX was used as a stable reference dye to correct for spectral baseline shift due to either solvent evaporation or instrument instability. About 200 μL of the solution was used to record the absorption spectrum of the dye at room temperature on a Beckman DU 65i spectrophotometer. This spectrum was taken as the spectrum at 99°C at time zero. The remaining solution was dispensed into 12 separate PCR vials at 50 μL per vial. The PCR vials containing the solution were placed in the heat block of an iCycler IQ preheated to 99°C. Four vials each time were taken out at time 0.5, 1.0 and 3.0 hour, respectively, and pooled into a UV/Vis cuvette to record the absorption spectrum at room temperature.

## Authors' contributions

FM designed the molecular structure of the EvaGreen dye and experiments to test the binding affinities for DNA and inhibition effect of the dye. WYL contributed to the design, synthesis and initial characterization of the dye. XX designed and performed the binding assays and PCR assays. All authors provided their expertise and crucial insights which led to the design concept of this optimal qPCR dye. All authors contributed, read and approved the final manuscript.
